# Effects of Mulch Type, Plant Cultivar, and Insecticide Use on Sweet Potato Whitefly Population in Chili Pepper

**DOI:** 10.1155/2020/6428426

**Published:** 2020-09-28

**Authors:** Andi Nasruddin, Nurariaty Agus, Alam Saubil, Jumardi Jumardi, Burhanuddin Rasyid, Andry Siriniang, Andi Dirham Nasruddin, Firdaus Firdaus, Ahwiyah Ekawaty Said

**Affiliations:** ^1^Department of Plant Pest and Disease, Faculty of Agriculture, Hasanuddin University, Makassar 90245, South Sulawesi Province, Indonesia; ^2^Ministry of Food Crop, Horticulture and Animal Husbandry, Mamuju 91512, West Sulawesi Province, Indonesia; ^3^Department of Soil Science, Faculty of Agriculture, Hasanuddin University, Makassar 90245, South Sulawesi Province, Indonesia; ^4^Faculty of Agriculture, Universitas Islam, Makassar 90245, South Sulawesi Province, Indonesia

## Abstract

One of the most devastating pests of chili pepper is the sweet potato whitefly (SPW), *Bemisia tabaci* (Gennadius) (Hemiptera: Aleyrodidae). It sucks plant sap, emits honeydew on which sooty mold fungi grow, and transmits the pepper yellow leaf curl Indonesia virus (PepYLCIV), the most damaging viral disease of chili in Indonesia. Farmers rely mainly on insecticide to control the insect with two to three sprays in a week. To reduce the insecticide use, an integrated approach needs to be developed. Therefore, the current study evaluated the effectiveness of the integration of reflective mulch, host plant resistance, and insecticide use for managing the sweet potato whitefly on the chili pepper. In 2018, a complete randomized block design was used in three separate trials to assess the effects of mulch, cultivar, or insecticide application frequency on the numbers of whitefly eggs, nymphs, and adults. In 2019, a split-split plot design was used to evaluate the effects of the integration of mulch, cultivar, and insecticide application frequency on the numbers of whitefly eggs, nymphs, and adults. The results showed that the reflective silver mulch had significantly lower numbers of whitefly eggs, nymphs, and adults, in comparison to the rice straw mulch and bare ground treatments. Chili plants cv. Bara was more resistant than Bhaskara against *B. tabaci* in the field; however, in the no-choice trial, no significant difference was detected between both cultivars. Insecticide applications twice per week and once per week were equally effective in controlling the whitefly on the susceptible cultivar (Bhaskara). Overall, the integration of reflective mulch, resistant cultivar, and insecticide application every two weeks effectively suppressed *B. tabaci* populations on the chili pepper. This approach could substantially reduce the number of insecticide applications from twice per week (commonly practiced by chili farmers in the area) to one application only in two weeks.

## 1. Introduction

Chili pepper (*Capsicum frutescens* L.) is an important horticultural crop worldwide with high nutritional content. The plant also has high economic values [1]; hence, many smallholder farmers in Indonesia depend on this crop for their welfare. However, chili pepper cultivation is constantly challenged by numerous limiting factors and one of the most important limiting factors is the sweet potato whitefly (SPW), *Bemisia tabaci* (Genn.) (Hemiptera: Aleyrodidae) [2]. The whitefly can inflict direct damage to chili plants by sucking the plant sap when feeding, causing distorted young leaves and silver chlorotic spots on the leaves. Besides that, it also indirectly damages plants by emitting honeydew on the leaf surface on which sooty mold fungi grow producing black film covering the leaf surface that reduces the efficiency of photosynthesis [3, 4]. Another important indirect damage caused by the whitefly on chili pepper is the transmission of viral diseases on the crop, including the pepper yellow leaf curl Indonesia virus (PepYLCIV, Geminiviridae) [5], the most devastating viral disease on chili in Indonesia that can cause total yield loss to plants [6, 7].

Chili growers rely heavily on insecticide use to control the SPW with a high application frequency of two to three sprays in a week [8]. The practice can trigger the insect to develop resistance against insecticides used. The pest has developed resistance against pyrethroids, imidacloprid, and pyriproxyfen [9–12]. To gain a satisfactory control of the resistant pest, farmers use higher spray dosages than the recommended rates and/or spray their plants more frequently. This, of course, exacerbates the potential negative impacts of the insecticide use on the consumers and the environment. To reduce insecticide use, an integrated approach should be developed to control the SPW on chili pepper, by incorporating different control measures.

The use of plastic mulch and organic mulch, specifically from rice straw, is now becoming common practice used by chili pepper growers in many parts of Indonesia. The mulch is used mainly to control weed, maintain soil moisture, and increase yields [13]. Besides that, reflective plastic mulch and organic mulch have been reported effective in suppressing aphid, thrips, and, whitefly populations because the UV light reflected by the mulches deters the pests from landing on plants during host searching [14].

In addition, the whitefly can also be effectively controlled by using resistant cultivars [15]. Plant resistance mechanisms against the whitefly can be divided into three categories: nonpreference/antixenosis, antibiosis, and tolerance as described by [16]. Insect preference for feeding and laying eggs can be affected by leaf color and structure [17], leaf pubescence [18, 19], cuticle thickness [20], and the presence of repelling and attracting metabolites [21]. The antibiosis mechanism is expressed by plants producing some secondary metabolites, acting as antibiotic substances that negatively affect insect growth [22]. However, plants that have a tolerance resistance mechanism against insect pests produce a higher yield than susceptible plants under similar infestation pressure to compensate for the yield loss due to the pest damage [23].

Therefore, we hypothesized that the combination of mulch and resistant cultivar would be able to effectively suppress the SPW populations on chili pepper with reduced use of an insecticide. To our knowledge, no such research has been reported in South Sulawesi Province of Indonesia; hence, the objective of the current study was to evaluate the effectiveness of the integration of mulch, host plant resistance, and insecticide application frequency for managing the sweet potato whitefly on the chili pepper.

## 2. Materials and Methods

### 2.1. Study Site

Studies were conducted at the Agriculture Experiment Station, Faculty of Agriculture, Hasanuddin University (5°07'42” S, 119°28'47” E), Makassar, Indonesia, from June to November 2018 and from April to November 2019. The station is located about 7 m above the sea level with an average temperature of 27.5°C, ranging from 22.5 to 32.5°C, and an average rainfall of 261 mm/month, ranging from 15 mm to 734 mm/month.

### 2.2. Field Experiment 1, 2018

Three separate trials were conducted to determine (1) the effect of mulch type on the numbers of whitefly eggs, nymphs, and adults, (2) the effect of insecticide application frequency on the numbers of whitefly eggs, nymphs, and adults, and (3) the effect of cultivar on the numbers of whitefly eggs, nymphs, and adults. For the mulch and the insecticide trials, chili pepper (*C. frutescens*) cv. Bhaskara was used. In each trial, treatments were arranged in a complete randomized block design with four replications. Each replication was a plot (four rows wide and four meters long) of chili pepper with a planting space of 60 cm between rows and 50 cm within a row. For the mulch experiment, treatments were reflective silver mulch, rice straw mulch, and bare ground. For the insecticide experiment, insecticide Endure 120SC (spinetoram, Dow Agrosciences) was used and the treatments consisted of four insecticide application frequencies: twice in a week (local farmers' practice), once in a week, once in two weeks, and control (plant sprayed with water). For the cultivar experiment, treatments were two cultivars of chili pepper, Bara and Bhaskara. These two cultivars were selected because previous experiment results indicated that Bara was more resistant than Bhaskara against the whitefly adults in the field.

For all trials, the soil was cultivated using a hand tractor and seedbeds were prepared using hoes. Planting beds were raised 25 cm and there was a 2 m bare space between beds. All plant husbandry followed the local recommendations for chili pepper cultivation. One week before seedlings were transplanted, chicken manure compost was applied at a rate equivalent to 2 tons per ha. Three-week-old seedlings were transplanted on 25 June 2018, and two weeks later, the plants were fertilized with NPKS (15 : 15 : 15 : 10) (Phonska, Petrokimia Gresik) at a rate of 50 kg/ha. The fertilizer was applied every 10 days until the plants were blooming. When the plants started blooming, they were fertilized with a mixture of NPKS and SP36 (1 : 1) at a rate of 50 kg/ha every 10 days. Plants were irrigated as needed using a sprinkler system. To determine the weekly populations of adult whitefly, five plants per plot were randomly selected from two middle rows. From each plant sample, three leaves (1 lower leaf, 1 middle leaf, and 1 upper leaf) were observed by slowly turning the leaf and all adults present were counted. The same leaves were picked up and placed inside of separated Ziploc bags and then brought to the laboratory of Insect in Relation to Plant Disease, Hasanuddin University, for egg and nymph counts under a dissecting microscope at 200x (Zeiss Stemi DV-4). The eggs and nymphs were counted by placing a card with a 1 × 1 cm hole onto the lower leaf surface, next to the midrib in the center of the leaf. Results were recorded as the number of eggs or nymphs per cm^2^ of the leaf surface. The insect counts were performed weekly starting from 3 weeks after transplanting for five counts.

### 2.3. Cage Pot Experiment, 2018

A greenhouse trial was conducted to determine the whitefly populations on two chili cultivars, Bara and Bhaskara, in a no-choice cage environment. Each cultivar treatment had five replications. Each replication consisted of a cage (45 cm wide × 45 cm long × 75 cm high) made of an aluminum frame covered with a fine cloth. Five three-week-old plants grown in separate polybags were placed in each cage. The test plants were fertilized and watered, as necessary. Ten adult whiteflies were released into each cage. The numbers of eggs, nymphs, and adults were determined seven days after the whitefly release and every seven days thereafter for a total of five observations. For egg, nymph, and adult counts, the procedures described before were followed.

### 2.4. Field Experiment, 2019

The field trial was conducted using a split-split plot design with three factors, consisting of four insecticide frequency treatments: twice per week, once per week, once per two weeks, and control (plants sprayed with water); three mulch treatments: reflective silver plastic mulch, rice straw mulch, and bare ground; and two varietal treatments: Bara and Bhaskara. Four blocks were established with insecticide treatments as the main plots, mulch as the subplot, and cultivar as sub-subplot. Each mulch treatment had three replications and each replication had two rows of Bara and two rows of Bhaskara cultivars (10 plants for each cultivar). Planting spacing used was 60 cm between rows and 50 cm between plants in a row. Insecticide Alika 247ZC (lambda cyholothrin106 g/l + thiamethoxam 141 g/l) (Syngenta, Jakarta) was applied according to the prescribed treatments, using the full recommended rate of 0.4 ml/liter of water with a spray volume of 400 liters per ha. The insecticide was applied using a battery-powered backpack sprayer (Agropower 3WBD-16LA; Taizhou Taike Electronics Co., Ltd. Zhejiang, China). For the mulch treatment, reflective polyethylene plastic mulch 2 m wide and 0.08 mm thick (CV. Sumber Tani, Jember) was used to cover the plots and stretched by using bamboo stakes on plot edges. The plant husbandry followed the local recommendations for chili pepper cultivation as described before. To determine the weekly populations of egg, nymph, and adult of the whitefly, the procedures described before were applied. All experimental plots were weekly applied with a fungicide to protect the plants from anthracnose disease caused by fungus *Colletotrichum* spp.

### 2.5. Statistical Analysis

For the cultivar experiments, treatment counts were log-transformed before being compared using a Student's paired comparison *t*-test. For the other experiments, count data were log-transformed and then subjected to ANOVA before treatment means were separated using Tukey's post hoc test at *p*=0.05 (Jamovi, version 1.2.17, 2020).

## 3. Results

### 3.1. Field and Greenhouse Experiments, 2018

There were significant differences among the mulch treatments in the numbers of eggs, nymphs, and adults (*F* = 3.57, *p* < 0.05; *F* = 5.18, *p* < 0.05, and *F* = 14.50, *p* < 0.01, respectively; Table 1). The lowest numbers of eggs, nymphs, and adults were found in the reflective mulch, which were significantly lower than those in the bare ground and rice straw mulch. However, the highest numbers of the three variables were found in the rice straw mulch treatment, which were significantly higher than the bare ground.

In the free choice field experiment (2018), there were significant differences detected among the varietal treatments in the numbers of eggs, nymphs, and adults (*t* = 4.90, *p* < 0.05; *t* = 3.32, *p* < 0.05, and *t* = 5.20, *p* < 0.05, respectively; Table 2). The numbers of eggs, nymphs, and adults found in Bhaskara were significantly higher than those in Bara. However, in the no-choice greenhouse experiment, there were no significant differences detected between both cultivars in all variables measured.

There were significant differences among the application frequency treatments in the numbers of eggs, nymphs, and adults of *B. tabaci* (*F* = 18.41, *p* < 0.01; *F* = 38.23, *p* < 0.01, and *F* = 27.20, *p* < 0.01, respectively; Table 3). All application frequency treatments were significantly lower than the control in all variables measured. For the numbers of eggs, nymphs, and adults, twice and once per week treatments were not significantly different from each other but both were significantly lower than once per two weeks treatment. However, for the adult numbers, there were no significant differences detected among the frequency treatments.

### 3.2. Field Experiment, 2019

There were significant differences among the combined treatments of mulch and insecticide in the numbers of eggs, nymphs, and adults of *B. tabaci* (*F* = 11.9, *p* < 0.05; *F* = 6.17, *p* < 0.05, and *F* = 9.10, *p* < 0.05, respectively; Table 4). For reflective mulch, the numbers of eggs, nymphs, and adults on plants sprayed twice/week, once/week, and once/two weeks were not significantly different from each other but significantly lower than the control. For rice straw mulch and bare ground treatments, the numbers of eggs, nymphs, and adults on plants sprayed twice/week and once/week were not significantly different from each other but significantly lower than the plants sprayed once/2 weeks and the control. Overall, for reflective mulch, one insecticide application in two weeks was as effective as two applications per week. On the other hand, for straw and bare ground, two sprays in a week were as effective as one spray in a week; however, straw mulch with two sprays in a week still had significantly higher populations than the reflective mulch with one spray per 2 weeks.

There were significant differences among the combined treatments of mulch and cultivar in the numbers of eggs, nymphs, and adults of *B. tabaci* (*F* = 7.76, *p* < 0.05; *F* = 8.10, *p* < 0.05, and *F* = 9.09, *p* < 0.05, respectively; Table 5). Treatment of reflective mulch and Bara had the lowest numbers of eggs, nymphs, and adults in comparison to the other treatments. The highest numbers of eggs and nymphs were found on rice straw mulch and Bhaskara which were not significantly different from those on the bare ground and Bhaskara. However, the number of adults on rice straw mulch and Bhaskara was significantly higher than that on the bare ground and Bhaskara. The combined treatments of reflective mulch and Bhaskara, rice straw mulch and Bara, and bare ground and Bara were not significantly different in the numbers of eggs and nymphs. However, the number of adults on rice straw mulch and Bara was lower than the number of adults on the bare ground and Bara treatment but they were not significantly different from each other.

There were significant differences among the combined treatments of cultivar and insecticide in the numbers of eggs, nymphs, and adults of *B. tabaci* (*F* = 7.26, *p* < 0.05; *F* = 6.97, *p* < 0.05, and *F* = 8.10, *p* < 0.05, respectively; Table 6). For Bara, the numbers of eggs, nymphs, and adults on plants sprayed twice/week, once/week, and once/two weeks were not significantly different from each other but significantly lower than the control. For Bhaskara, the numbers of eggs, nymphs, and adults on plants sprayed twice/week and once/week were not significantly different from each other but significantly lower than the plants sprayed once/2 weeks and the control. Overall, the insect populations on Bara sprayed once in two weeks were significantly lower than those on Bhaskara sprayed twice in a week.

## 4. Discussion

Insecticides are used intensively by chili growers, 2–3 sprays in a week, to control *B. tabaci* on chili pepper [8]. The excessive use of insecticide could pose detrimental impacts on consumers' health, especially if the product is consumed in a raw state like chili pepper, and to the environment. In the current study, the combined effects of different types of mulch, insecticide application frequencies, and chili cultivars on the *B. tabaci* populations were evaluated. We intended to develop a treatment combination that is effective yet with minimal use of an insecticide.

From the single factor trials in 2018, reflective silver mulch had significantly lower numbers of eggs, nymphs, and adults, compared to bare ground, and rice straw mulch treatments. Ultraviolet light reflected from the mulch deterred the whitefly adults to land and lay eggs on the plants. Our data agreed to the previous reports that fewer adults alight on the plants with reflective mulch; in consequence, fewer eggs were laid and fewer nymphs emerged from the eggs [14] (Table 1). In contrast, the highest numbers of eggs, nymphs, and adults were found on the plants with rice straw mulch, which were about 6.7, 6.6, and 7.4 times more than those in the reflective mulch experiment. This is probably due to the fact that the combination of the leaf color (light green) of the Bhaskara cultivar and the yellow color of the rice straw was more attractive than the bare ground color to the whitefly. Adults of *B. tabaci* are strongly attracted to the yellow surface [24, 25], and because of this, the insect is more attractive to young leaves, which are yellower, than older leaves [26].

In the field (free choice) experiment, the average numbers of eggs, nymphs, and adults were significantly higher on Bhaskara than those on Bara. Bhaskara seemed to be more attractive to the whitefly adults because it has bigger and lighter green leaves than Bara. Insect preference for host selection to feed and lay eggs is affected by leaf color and structure [27]. Adult whiteflies are strongly attracted to the yellow surface [25]. Thus, Bara resistance against the whitefly was probably based on antixenosis mechanism. This is confirmed by the no-choice greenhouse experiment results showing that no significant differences were detected between both cultivars in the numbers of eggs, nymphs, and adults.

Insecticide application of twice per week as practiced by local chili farmers was not necessary because the application of once per week treatment was enough to provide satisfactory control of whitefly on the susceptible cultivar, Bhaskara. This is in line with [27] reporting that insecticide application in every six days is effective in controlling the silverleaf whitefly. The farmers' practice of two applications in seven days [8] is most likely based on their determination of saving their crops, so they apply insecticide as frequently as possible. In addition, since chili pepper has a high economic value [1], the farmers consider that the insecticide application costs are economically justifiable.

When reflective mulch was combined with insecticide, satisfactory control of *B. tabaci* could be achieved with reduced application frequency from twice per week to once in two weeks. The results even suggested that the reflective mulch allowed farmers to apply insecticide less than the recommended application frequency of once every six days when mulch is not used [27]. For the treatment combination of mulch and cultivar, the whitefly populations were consistently and significantly lower on Bara than on Bhaskara in all types of mulch. Reflective mulch and Bara had the lowest numbers of eggs, nymphs, and adults. This is in agreement with [28] reporting that the combination of reflective mulch and resistant accession significantly reduced the whitefly population. Our results also showed that when cultivar and insecticide were combined, for the resistant cultivar (Bara), one insecticide application in two weeks was as effective as two applications in a week. However, for the susceptible cultivar (Bhaskara), two applications per week were still significantly less effective than Bara with one application in two weeks.

## 5. Conclusion

The results of the current study revealed that the integration of reflective silver mulch, resistant cultivar (Bara), and insecticide application every two weeks effectively suppressed the sweet potato whitefly populations on the chili pepper. The integrated approach could substantially reduce the number of insecticide applications from twice per week, which is commonly practiced by chili farmers in the area, to one application per two weeks. Further studies should be conducted to determine the effects of the treatments on the PepYLCIV spread by the SPW in the field.

## Figures and Tables

**Table 1:** Mean numbers of whitefly eggs, nymphs, and adults for mulch type treatments, 2018.

Mulch	Mean numbers of whiteflies (±SEM)		
	Eggs per 1 cm^2^ leaf	Nymphs per 1 cm^2^ leaf	Adults per plant
Reflective silver mulch	3.47 ± 0.50 *a*	3.17 ± 0.27 *a*	2.24 ± 0.12 *a*
Rice straw mulch	23.15 ± 2.41 *c*	16.82 ± 1.46 *b*	21.01 ± 1.68 *c*
Bare ground	17.19 ± 1.38 *b*	16.62 ± 1.67 *c*	12.20 ± 0.42 *b*

Means in a column followed by different letters are significantly different from each other (*p* < 0.05), according to Tukey's post hoc test.

**Table 2:** Mean numbers of whitefly eggs, nymphs, and adults on chili cultivar treatments in field and greenhouse experiments, 2018.

Cultivar	Mean numbers of whiteflies (±SEM)		
	Eggs per 1 cm^2^ leaf	Nymphs per 1 cm^2^ leaf	Adults per plant
*Field Experiment*			
Bara	2.25 ± 0.25 *a*	1.75 ± 0.25 *a*	1.25 ± 0.25 *a*
Bhaskara	4.25 ± 0.48 *b*	3.25 ± 0.48 *b*	2.75 ± 0.29 *b*

*Greenhouse Experiment*			
Bara	17.4 ± 1.7	12.7 ± 1.5	24.6 ± 1.1
Bhaskara	15.9 ± 3.1	14.2 ± 1.6	26.3 ± 2.5

Means in a column within the same type of experiment followed by different letters are significantly different from each other (*p* < 0.05), according to the paired comparison *t-*test.

**Table 3:** Mean numbers of whitefly eggs, nymphs, and adults for insecticide application frequency treatments, 2018.

Insecticide application	Mean numbers of whiteflies (±SEM)		
	Eggs per 1 cm^2^ leaf	Nymphs per 1 cm^2^ leaf	Adults per plant
Twice per week	2.55 ± 0.39 *a*	2.61 ± 0.29 *a*	2.17 ± 0.33 *a*
Once per week	2.96 ± 0.51 *a*	2.87 ± 0.46 *a*	2.16 ± 0.32 *a*
Once per two weeks	6.78 ± 0.25 *b*	5.49 ± 0.25 *b*	3.41 ± 0.94 *a*
Control	14.54 ± 3.91 *c*	10.34 ± 2.40 *c*	12.61 ± 1.24 *b*

Means in a column followed by different letters are significantly different from each other (*p* < 0.05), according to Tukey's post hoc test.

**Table 4:** Influence of insecticide and mulch treatments on the average numbers of whitefly eggs, nymphs, and adults across cultivars, 2019.

Mulch	Insecticide application	Mean numbers of whiteflies (±SEM)		
		Eggs per 1 cm^2^ leaf	Nymphs per 1 cm^2^ leaf	Adults per plant
Reflective silver mulch	Twice/week	1.14 ± 0.09 *a*	1.17 ± 0.09 *a*	0.86 ± 0.03 *a*
Reflective silver mulch	Once/week	1.18 ± 0.48 *a*	1.6 ± 0.27 *a*	0.95 ± 0.07 *a*
Reflective silver mulch	Once/2 weeks	2.21 ± 0.65 *a*	1.64 ± 0.21 *a*	0.99 ± 0.17 *a*
Reflective silver mulch	No spray (control)	4.90 ± 1.05 *b*	5.20 ± 1.25 *b*	3.20 ± 0.30 *c*
Rice straw mulch	Twice/week	5.11 ± 0.60 *b*	4.40 ± 1.58 *b*	1,59 ± 0.35 *b*
Rice straw mulch	Once/week	7.96 ± 1.92 *b*	3.98 ± 1.31 *b*	1.68 ± 0.40 *b*
Rice straw mulch	Once/2 weeks	10.17 ± 1.62 *c*	6.13 ± 1.78 *b*	3.73 ± 0.38 *c*
Rice straw mulch	No spray (control)	15.66 ± 1.4 *d*	13.94 ± 2.89 *d*	12.92 ± 0.63 *e*
Bare ground	Twice/week	3.68 ± 0.40 *b*	3.15 ± 0.54 *b*	1.18 ± 0.10 *b*
Bare ground	Once/week	5.48 ± 0.28 *b*	3.62 ± 0.71 *b*	1.34 ± 0.14 *b*
Bare ground	Once/2 weeks	13.42 ± 1.81 *c*	5.35 ± 1.04 *b*	3.99 ± 0.60 *c*
Bare ground	No spray (control)	18.96 ± 2.06 *d*	9.20 ± 1.85 *d*	8.53 ± 0.60 *d*

Means in a column followed by different letters are significantly different from each other (*p* < 0.05), according to Tukey's post hoc test.

**Table 5:** Influence of mulch and cultivar treatments on the average numbers of whitefly eggs, nymphs, and adults across insecticide treatments, 2019.

Mulch	Cultivar	Mean numbers of whiteflies (±SEM)		
		Eggs per 1 cm^2^ leaf	Nymphs per 1 cm^2^ leaf	Adults per plant
Reflective mulch	Bhaskara	2.40 ± 0.45 *a*	1.49 ± 0.17 *a*	0.91 ± 0.09 *a*
Reflective mulch	Bara	4.87 ± 0.96 *b*	3.33 ± 0.74 *b*	1.49 ± 0.19 *b*
Rice straw mulch	Bhaskara	5.59 ± 0.64 *b*	2.67 ± 0.51 *b*	1.05 ± 0.09 *a*
Rice straw mulch	Bara	13.86 ± 1.32 *c*	11.55 ± 1.75 *c*	8.91 ± 0.37 *d*
Bare ground	Bhaskara	7.31 ± 0.87 *b*	2.62 ± 0.39 *b*	1.32 ± 0.19 *b*
Bare ground	Bara	13.46 ± 1.89 *c*	8.04 ± 0.96 *c*	5.33 ± 0.27 *c*

Means in a column followed by different letters are significantly different from each other (*p* < 0.05), according to Tukey's post hoc test.

**Table 6:** Influence of cultivar and insecticide application frequency treatments on the average numbers of whitefly eggs, nymphs, and adults across mulches, 2019.

Cultivar	Insecticide	Mean numbers of whiteflies (±SEM)		
		Eggs per 1 cm^2^ leaf	Nymphs per 1 cm^2^ leaf	Adults per leaf
Bara	Twice/week	2.53 ± 0.35 *a*	1.27 ± 0.11 *a*	0.84 ± 0.04 *a*
Bara	Once/week	2.37 ± 0.61 *a*	1.28 ± 0.11 *a*	0.82 ± 0.84 *a*
Bara	Once/two weeks	3.25 ± 0.30 *a*	1.62 ± 0.18 *a*	0.85 ± 0.04 *a*
Bara	Control	10,10 ± 0.84 *c*	4.99 ± 0.48 bc	2.10 ± 0.17 *c*
Bhaskara	Twice/week	4,10 ± 0.40 *b*	4.52 ± 1.04 *b*	1.58 ± 0.22 *b*
Bhaskara	Once/week	6.71 ± 1.63 *b*	5.09 ± 0.88 *b*	1.76 ± 0.25 *b*
Bhaskara	Once/two weeks	13.11 ± 1.53 *d*	7.13 ± 1.22 *c*	1.85 ± 0.22 bc
Bhaskara	Control	18.99 ± 1.89 *e*	13.92 ± 2.24 *d*	3.78 ± 0.42 *d*

Means in a column followed by different letters are significantly different from each other (*p* < 0.05), according to Tukey's post hoc test.

## Data Availability

All data supporting the findings of this study are included within the article.
